# Health-related quality of life impact of scabies in the Solomon Islands

**DOI:** 10.1093/trstmh/trab096

**Published:** 2021-06-28

**Authors:** Susanna J Lake, Daniel Engelman, Oliver Sokana, Titus Nasi, Dickson Boara, Michael Marks, Margot J Whitfeld, Lucia Romani, John M Kaldor, Andrew C Steer, Natalie Carvalho

**Affiliations:** Tropical Diseases Research Group, Murdoch Children's Research Institute, Melbourne, Victoria, Australia; Department of Paediatrics, University of Melbourne, Melbourne, Victoria, Australia; Melbourne Children's Global Health, Royal Children's Hospital, Melbourne, Victoria, Australia; Tropical Diseases Research Group, Murdoch Children's Research Institute, Melbourne, Victoria, Australia; Department of Paediatrics, University of Melbourne, Melbourne, Victoria, Australia; Melbourne Children's Global Health, Royal Children's Hospital, Melbourne, Victoria, Australia; Ministry of Health and Medical Services, Solomon Islands; Ministry of Health and Medical Services, Solomon Islands; Ministry of Health and Medical Services, Solomon Islands; London School of Hygiene and Tropical Medicine, London, UK; Hospital for Tropical Diseases, London, UK; St Vincent's Hospital, University of New South Wales, Sydney, NSW, Australia; Kirby Institute, University of New South Wales, Sydney, NSW, Australia; Kirby Institute, University of New South Wales, Sydney, NSW, Australia; Tropical Diseases Research Group, Murdoch Children's Research Institute, Melbourne, Victoria, Australia; Department of Paediatrics, University of Melbourne, Melbourne, Victoria, Australia; Melbourne Children's Global Health, Royal Children's Hospital, Melbourne, Victoria, Australia; Melbourne School of Population and Global Health, University of Melbourne, Melbourne, Victoria, Australia

**Keywords:** Scabies, Quality of life, Solomon Islands

## Abstract

**Background:**

Scabies causes intense itching and skin lesions. A small number of studies have shown that scabies impacts health-related quality of life (HRQoL), but no studies have been conducted in the Pacific region. We assessed the impact of scabies on HRQoL in a high-prevalence setting using the Children's Dermatology Life Quality Index (CDLQI) and Dermatology Life Quality Index (DLQI). We also assessed the validity of these tools in a Pacific Island population.

**Methods:**

The study was conducted in the Solomon Islands. Participants with and without skin disease were randomly selected. HRQoL indices were scored on a scale of 0–30.

**Results:**

We surveyed 1051 adults (91 with scabies) and 604 children (103 with scabies). Scabies had a small impact on HRQoL, with a median DLQI score of 2 (interquartile range [IQR] 0–6) and a CDLQI score of 2 (IQR 0–4). Scores increased linearly with severity. The greatest impact on QoL was due to itch, sleep disturbance and impacts on education and employment.

**Conclusions:**

Scabies has a small but measurable impact on HRQoL. The DLQI and CDLQI scores were discriminated between the skin-related QoL of patients with scabies and the control group, indicating that these tools are appropriate to measure skin-related QoL in the Solomon Islands.

## Introduction

Scabies is caused by an infestation with the microscopic mite *Sarcoptes scabiei* var. *hominis*. This parasitic mite burrows into the skin and reproduces, causing intense itch and skin lesions. These lesions can become infected with bacteria, leading to impetigo, invasive bacterial infections and serious immune-mediated complications.^1^ Scabies is estimated to cause 455 million annual incident cases globally, with the vast majority of cases occurring in disadvantaged populations.^2^ According to the 2015 Global Burden of Disease Study, scabies was responsible for 0.21% of disability-adjusted life years (DALYs) of all 315 conditions included in the study.^3^ The disability weight assigned to scabies was 0.027 on a scale of 0 (perfect health)–1 (death).^3^ This estimate of DALYs and disability weight accounted for the primary effects of scabies (itch and skin lesions) but did not consider the secondary effects of bacterial infection, nor mortality related to crusted scabies or bacterial complications, including invasive bacterial infections and immune-mediated diseases, or broader impacts of scabies on quality of life.

Scabies contributes to morbidity as a result of both the clinical pathology and emotional impact of the disease.^4^ Scabies may impact a person in a number of ways: the intense itch can affect sleep and concentration at school or work and the appearance of lesions and the highly infectious nature of the disease can lead to stigma.^5^ Secondary complications can result in hospitalisation, cause long-term disability and may even be fatal.^1^ Four previous studies have investigated health-related quality of life (HRQoL) impacts in individuals with scabies. Studies were conducted in Brazil (n=105, children and adults), China (n=96, adults only), India (n=102, children and adults) and Liberia (n=124, children and adults).^4, 6–8^ All four studies used the Dermatology Life Quality Index (DLQI) or a modified version of the DLQI, three used the Children's Dermatology Life Quality Index (CDLQI) for paediatric participants and one used the Family Dermatology Life Quality Index (FDLQI). These studies found that scabies had a small to moderate impact on HRQoL and that the impact was greater in adults. The domains most impacted by scabies were embarrassment and shame and work and study.

HRQoL is a multidimensional concept that captures physical, emotional, psychosocial and social aspects of well-being and can be measured using either generic or disease-specific instruments.^9^ Generic instruments measure domains such as mobility and self-care and can be used across different disease areas. Dermatology-specific instruments may be more sensitive to the impact of skin disease on HRQoL.^10,11^ The DLQI and CDLQI are two examples of validated surveys that have been used extensively for skin conditions including psoriasis, atopic eczema and acne.^12^ While the DLQI is most commonly used, its limitations include its focus on disability, cultural equivalence issues, item bias and structure.^13^ The DLQI is not a preference-based tool, but mapping studies exist to convert DLQI scores to a preference-based valuation that is relevant when assessing the cost-effectiveness of interventions for scabies.^14^

### Objectives

Our aim was to assess the level and domains of impact of scabies on HRQoL in children and adults. We also aimed to establish construct validity for and suitability of the use of the DLQI and CDLQI in a Pacific Island country.

## Methods

### Setting

This study was conducted in the Western Province of Solomon Islands, a Melanesian country in the South Pacific comprised of >900 islands with a population of approximately 700 000 people.^15^ The most commonly spoken languages are English and Pijin.^16^ Solomon Islands is classified as a least-developed country, ranking 153 of 189 countries on the Human Development Index.

This study was conducted as part of the Regimens of Ivermectin for Scabies Elimination (RISE) trial (ACTRN12618001086257), a cluster-randomised non-inferiority trial of one versus two doses of ivermectin for the control of scabies.^17^ All participants in the study were offered treatment for scabies as part of a mass drug administration campaign. The RISE trial was conducted in 20 remote villages, each with a population of 200–400 people. Most residents rely on subsistence agriculture and fishing. Three of the study villages have a basic health clinic staffed by nurses. Members of other villages travel by foot or canoe for their medical care. The prevalence of scabies in the study villages is 15%.^18^ Benzyl benzoate lotion is listed on the Solomon Islands Essential Medicines List and is available free of charge at health clinics for the treatment of scabies.

### Study design

We conducted a cross-sectional study using the DLQI and CDLQI instruments to assess the impact of scabies on HRQoL.^11^ All village residents were eligible to participate in the RISE trial and participants ≥4 y of age were eligible to take part in the HRQoL assessment. We aimed to select approximately one-third of all participants in the RISE trial to complete a DLQI or CDLQI questionnaire. The staff member administering the questionnaire randomly selected participants and aimed to stratify the random sample by gender and age group so that the sample was reflective of all RISE trial participants.

Nurses were trained to conduct clinical examinations of participants to assess for scabies and impetigo.^19^ All participants in the study were examined. The examination included history features of scabies as well as examination of exposed areas (upper and lower limbs in all participants and head and trunk in those <2 y of age). Examinations took place in an open setting in the village. Diagnosis was made using the 2020 International Alliance for the Control of Scabies (IACS) Consensus Criteria for the Diagnosis of Scabies.^20^ Impetigo was defined as sores with papular, pustular or ulcerative lesions surrounded by erythema or with crusts, pus or bullae.^18^ Secondarily infected scabies lesions, abscesses or cellulitis were also classified as impetigo for data analysis. The severity of both scabies and impetigo was classified by the number of lesions (very mild, 1–2 lesions; mild, 3–10 lesions; moderate, 11–50 lesions; severe, >50 lesions). Participants were considered free of skin disease if they had no rash (either scabies or non-scabies), itch, infected sores, cellulitis, ulcers, abscesses or boils.

### QoL measurement tools

The DLQI (participants ≥15 y of age) and CDLQI (participants 4–14 y of age) instruments were administered after a skin examination was completed. The questionnaires were administered by the same staff member so that questioning was consistent. The DLQI and CDLQI have not been validated in Solomon Islands Pijin and literacy rates in villages were too low for participants to self-administer the written questionnaire.^16^ The questionnaire was therefore conducted verbally in English with Pijin translation when required to provide clarification. Prior to commencing the study, the questions were translated into Pijin by the study team to ensure consistent clarification was provided. If the participant was unable to communicate in English or Pijin the questionnaire was not administered.

We conducted the DLQI and CDLQI among participants both with and without scabies or other skin conditions. The participants without scabies were considered as controls in order to establish the validity of the tools in this population, as there has been no validation of the questionnaire in Pacific Island countries. Participants free of skin conditions have been used as a control population to establish the construct validity of the questionnaire in other studies.^12,21^

Both the DLQI and CDLQI were scored on a 4-point Likert scale, with each of the 10 questions scored out of 3 points (0, ‘not at all’; 1, ‘a little’; 2, ‘a lot’; 3, ‘very much’), with the total score ranging from 0 to 30.^11^ The DLQI also includes a ‘not relevant’ answer, scored as 0. During the analysis, scores were banded using an established method to assess the level of impairment.^22^

### Analysis

DLQI and CDLQI data were linked to skin examination data to match survey responses to the clinical examination. The scores were non-normally distributed, so we calculated both median and mean (statistic most relevant for a cost-effectiveness analysis) scores for participants with scabies, impetigo, non-scabies skin disease and without skin disease. We compared scores according to the severity of scabies. We grouped children into 5-y age groups and adults into 20-y age groups. We analysed the proportion of responses of participants with scabies who had any impairment (a score of ≥1) in each of the domains. Eight questions in the DLQI questionnaire include the option of ‘not relevant’ as a response. A large number of ‘not relevant’ responses can impact the DLQI score.^23^ We assessed the proportion of ‘not relevant’ responses for each question and recalculated an overall adjusted mean score (DLQI-R) using a standard formula that removes the ‘not relevant’ items and adjusts the total score for the relevant items.^23^ We used the Wilcoxon rank sum (Mann–Whitney) test to assess the significance of the scores of participants with scabies and control groups with a skin disease other than scabies and no skin disease.

Study data were collected into a REDCap database using an Android device by the staff member conducting the questionnaire. Data were managed using REDCap electronic data capture tools hosted at Murdoch Children's Research Institute (Melbourne, Victoria, Australia). Data were analysed using Stata version 14.2 (StataCorp, College Station, TX, USA).

### Consent and ethical approval

Informed consent was required to participate in the study with confirmation of consent either written or with thumbprint. Participants were informed of the study aims and design through provision of verbal and written information. Consent was provided by a parent or guardian for those <18 y of age. The study was approved by the Solomon Islands Health Research and Ethics Review Board (HRE005/18) and the Royal Children's Hospital Melbourne Human Research Ethics Committee (38099A).

## Results

### Participants

We surveyed 1051 adults and 604 children (Table 1), representing 35.9% of the 4612 participants in the RISE trial ≥4 y of age. Among our sample, 91 adults and 103 children had scabies. The sample was largely representative of those enrolled in the RISE trial and the general population, although there was a lower proportion of younger children (ages 4–9 y) and male adults compared with the RISE trial (Table 1).

**Table 1.** Demographic characteristics of participants

Characteristics	Participants, n (%)	Scabies, n (%)	Other skin disease, n (%)	No skin disease, n (%)	All RISE trial participants, n (%)
CDLQI					
Gender					
Female	284 (47.0)	36 (35.0)	59 (43.4)	189 (51.8)	920 (48.7)
Male	320 (53.0)	67 (65.0)	77 (56.6)	176 (48.2)	968 (51.3)
Age (years)					
4–9	261 (43.2)	41 (39.8)	67 (49.3)	153 (41.9)	1082 (57.3)
10–14	343 (56.8)	62 (60.2)	69 (50.7)	212 (58.1)	806 (42.7)
Total	604	103	136	365	1888
DLQI					
Gender					
Female	665 (63.3)	55 (60.4)	136 (59.6)	474 (64.8)	1527 (56.1)
Male	386 (36.7)	36 (39.6)	92 (40.4)	258 (35.2)	1197 (43.9)
Age (years)					
15–19	152 (14.5)	16 (17.6)	34 (14.9)	102 (13.9)	383 (14.1)
20–39	481 (45.8)	47 (51.6)	94 (41.2)	340 (46.4)	1217 (44.6)
40–59	312 (29.7)	23 (25.3)	68 (29.8)	221 (30.2)	788 (28.9)
≥60	106 (10.1)	5 (5.5)	32 (14.0)	69 (9.4)	336 (12.3)
Total	1051	91	228	732	2724

### CDLQI/DLQI scores for participants with scabies

CDLQI scores for children and DLQI scores for adults were similar (children: median 2 [IQR 0–4], mean 2.8 [95% confidence interval {CI} 2.3 to 3.4]; adults: median 2 [IQR 0–6], mean 3.1 [95% CI 2.4 to 3.8]; Table 2). Both DLQI and CDLQI scores were significantly different for participants with scabies compared with participants without scabies (p<0.05). Among both children and adults, the median and mean scores on the CDLQI and DLQI corresponded to a ‘small effect’ of scabies on HRQoL. Participants commented that they were unlikely to seek treatment even if they had symptoms of scabies.

**Table 2.** Banding of CDLQI and DLQI scores

CDLQI	Scabies (n=103)	Other skin disease (n=136)
Median score (IQR)	2 (0–4)	0 (0–0)
Mean score (95% CI)	2.8 (2.3 to 3.4)	0.8 (0.4 to 1.1)
Score, %		
0	28.2	76.5
0–1, no effect	40.8	89
2–6, small effect	46.6	5.9
7–12, moderate effect	12.6	4.4
13–18, very large effect	0	0.7
19–30, extremely large effect	0	0
DLQI	Scabies (n=91)	Other skin disease (n=228)
Median score (IQR)	2 (0–6)	0 (0–1)
Mean score (95% CI)	3.1 (2.4 to 3.8)	0.8 (0.6 to 1.1)
Score, %		
0	33	74.6
0–1, no effect	49.5	85.1
2–5, small effect	23.1	9.2
6–10, moderate effect	24.2	4.4
11–20, very large effect	3.3	1.3
21–30, extremely large effect	0	0

CDLQI scores were higher in females than males (female children: median 3 [IQR 0.5–5.5], mean 3.3 [95% CI 2.2 to 4.3]; male children: median 2 [IQR 0–4], mean 2.6 [95% CI 1.9 to 3.2]), whereas DLQI scores were higher in adult males than adult females (adult females: median 1 [IQR 0–5], mean 2.8 [95% CI 1.9 to 3.7]; adult males: median 3.5 [IQR 0–6.5], mean 3.6 [95% CI 2.4 to 4.7]) (Table 3), however, the IQRs and CIs were overlapping across all groups. Scores among children were similar across age groups. In adults, the highest score was in those 20–39 y of age.

**Table 3.** CDLQI and DLQI scores for participants with scabies

Characteristics	Participants, n	CDLQI score	Participants, n	DLQI score
Gender				
Female, median (IQR)	36	3 (0.5–5.5)	55	1 (0–5)
Mean (95% CI)		3.3 (2.2 to 4.3)		2.8 (1.9 to 3.7)
Male, median (IQR)	67	2 (0–4)	36	3.5 (0–6.5)
Mean (95% CI)		2.6 (1.9 to 3.2)		3.6 (2.4 to 4.7)
Age (years)				
4–9, median (IQR)	41	2 (0–5)		
Mean (95% CI)		2.8 (2.0 to 3.6)		
10–14, median (IQR)	62	2 (0–4)		
Mean (95% CI)		2.9 (2.1 to 3.6)		
15–19, median (IQR)			16	1 (0–5)
Mean (95% CI)				2.8 (1.0 to 4.6)
20–39, median (IQR)			47	4 (0–7)
Mean (95% CI)				3.9 (2.8 to 4.9)
40–59, median (IQR)			23	1 (0–4)
Mean (95% CI)				2.0 (1.0 to 3.0)
≥60, median (IQR)			5	1 (0–3)
Mean (95% CI)				1.1 (0 to 5.2)
Location of scabies lesions^a^				
Upper limb, median (IQR)	103	2 (0–4)	87	2 (0–6)
Mean (95% CI)		2.8 (2.3 to 3.4)		3.1 (2.4 to 3.9)
Lower limb, median (IQR)	40	3 (1.5–5)	28	3.5 (0.5–6)
Mean (95% CI)		3.6 (2.6 to 4.5)		3.4 (2.2 to 4.7)
Head, median (IQR)	3	5 (3–5)		
Mean (95% CI)		4.3 (1.5 to 7.2)		
Trunk, median (IQR)	3	5 (3–7)		
Mean (95% CI)		5 (0 to 10)		
Skin condition present				
Scabies no impetigo, median (IQR)	96	2 (0–4)	86	1.5 (0–6)
Mean (95% CI)		2.8 (2.2 to 3.4)		3.0 (2.3 to 3.7)
Scabies and impetigo, median (IQR)	7	3 (0–5)	5	4 (0–6)
Mean (95% CI)		3.3 (0.6 to 6.0)		4.2 (0 to 9.9)
Impetigo no scabies, median (IQR)	34	0 (0–0)	21	0 (0–0)
Mean (95% CI)		0.4 (0 to 0.9)		0.1 (0 to 0.2)

a Participants may have lesions in more than one location.

**Figure 1.**
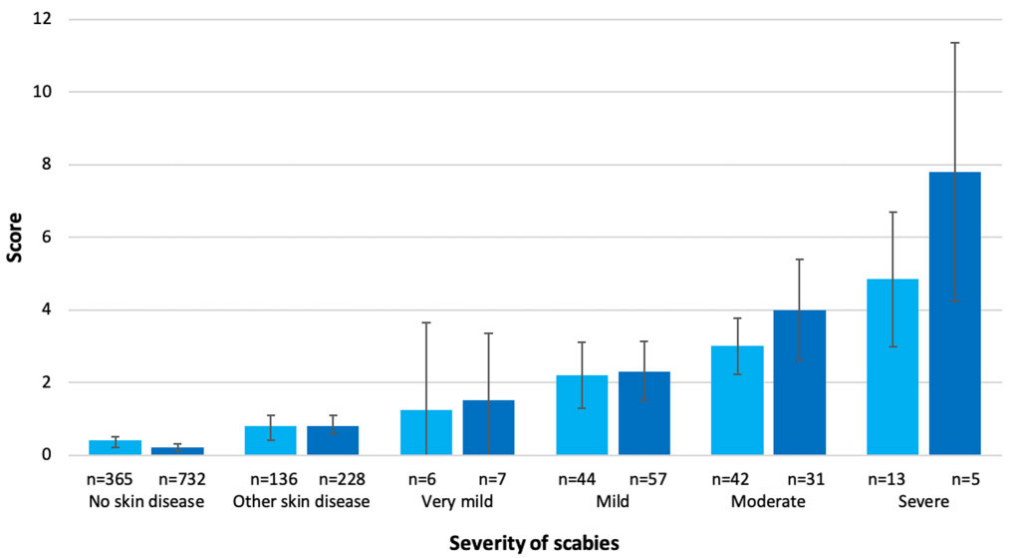
Mean score for the CDLQI and DLQI by scabies severity. Error bars indicate 95% CI: CDLQI, light blue; DLQI, dark blue).

Scores for both children and adults increased with the severity of scabies (children with severe scabies: mean 4.8 [95% CI 3.0 to 6.7]; adults with severe scabies: mean 7.8 [95% CI 4.2 to 11.4]) (Figure 1). In adults, the score for severe scabies corresponded to a moderate impact on HRQoL. However, in children with severe scabies the score still only corresponded to a small effect on HRQoL. Participants with scabies affecting the lower limbs, head or trunk had higher CDLQI and DLQI scores than those with scabies affecting the upper limbs (Table 3).

**Figure 2.**
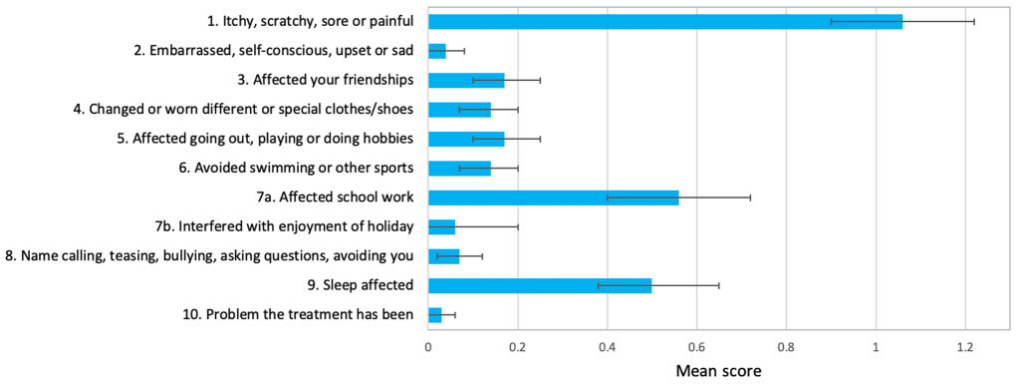
Mean CDLQI score for each question for participants with scabies (n=103). Each question scores between 0 and 3. Error bars indicate 95% CI.

The CDLQI items with the greatest impact on the total score were related to skin itchiness (mean 1.0; 32% of those with scabies reporting ‘quite a lot’ or ‘very much’), impact on schoolwork (mean 0.6) and sleep (mean 0.5; Figure2 and Supplementary Table 1.1). ‘Symptoms and feelings’ was the domain with the highest impact (70.9% scoring at least 1 point; Table 4) and this was due to the symptoms rather than feelings. The mean score for question 2 regarding feelings was the second lowest score (mean 0.04). Among children, there was a greater impact on girls in the personal relationships, leisure and school or holidays domains.

**Table 4.** Impairment^a^ of QoL in males and females with scabies by domain

Domain	CDLQI questions	Female (n=36), %	Male (n=67), %
Symptoms and feelings	1,2	75	68.7
Personal relationships	3, 8	33.3	14.9
Leisure	4, 5, 6	33.3	23.9
School or holidays	7	41.7	32.8
Sleep	9	41.7	37.3
Treatment	10	2.8	3.0
Domain	DLQI questions	Female (n=55), %	Male (n=36), %
Symptoms and feelings	1, 2	67.3	66.7
Daily activities	3, 4	34.5	44.4
Leisure	5, 6	23.6	41.7
Work and school	7	7.3	16.7
Personal relationships	8, 9	29.1	25.0
Treatment	10	0	5.6

a Impairment is equivalent to a score ≥1 in the domain.

**Figure 3.**
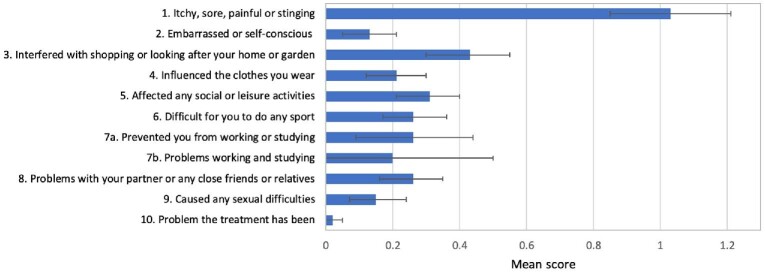
Mean DLQI score for each question for participants with scabies (n=91). Each question scores between 0 and 3. Error bars indicate 95% CI.

Among adults, the items with the greatest impact were also related to skin itchiness (mean 1.0; 33% of those with scabies reporting ‘a lot’ or ‘very much’) and shopping, housework and gardening (mean 0.4; Figure 3 and Supplementary Table 1.2). The domain with the highest impact among adults was ‘symptoms and feelings’ (67% scoring at least 1 point; Table 4). Similar to children, the ‘feelings’ question in this domain had a low score (mean 0.13). Among adults, the impact on leisure, work or school and daily activities domain was greater in males.

Most questions of the DLQI had a low proportion (<20%) of ‘not relevant’ responses, with the exception of impact on work or study, where 80.2% of participants with scabies responded ‘not relevant’. We calculated an adjusted mean DLQI score (DLQI-R) of 3.4 (95% CI 2.6 to 4.2) in participants with scabies, 0.2 (95% CI 0.1 to 0.3) in participants with no skin disease and 0.8 (95% CI 0.6 to 1.1) in participants with a skin disease other than scabies.

### Control group

Participants in the control group with no skin disease scored a median CDLQI/DLQI score of 0 (range 0–13) and the control group with a skin disease other than scabies scored a median CDLQI/DLQI score of 0 (range 0–15). There was a significant difference in both DLQI and CDLQI scores in each control group (p<0.05). Participants with a skin disease not diagnosed as scabies scored a mean CDLQI score of 0.8 (95% CI 0.4 to 1.1) and a mean DLQI score of 0.8 (95% CI 0.6 to 1.1) (Table 2). Participants with no skin disease scored a mean CDLQI score of 0.2 (95% CI 0.1 to 0.3) and a mean DLQI score of 0.2 (95% CI 0.1 to 0.3). In the ‘other skin disease’ group there were 16 adults and 28 children that had a typical scabies rash but did not meet the IACS diagnostic criteria for scabies due to a lack of supportive history features (itch or contact history). For these participants the mean CDLQI score was 0.5 (95% CI 0.2 to 0.8) and the mean DLQI score was 1.4 (95% CI 0 to –2.7).

## Discussion

We found that scabies affected HRQoL for both adults and children, with an average impact of a ‘small effect’ on a person's life. The impact on HRQoL increased with the severity of scabies. The results of a median score of 0 for the control group with no skin disease demonstrate that the DLQI and CDLQI show specificity in this population group. There is limited evidence for the use of the DLQI and CDLQI in Pacific Island countries and these results establish a construct validity in this setting.

Our study provides novel insights into the types of impacts scabies has on HRQoL in a population with a high prevalence of the disease and how this differs from other studies. The domain most affected was ‘symptoms and feelings’ as a result of itch, a common symptom of scabies. Among children in the study, scabies had an impact on schoolwork in more than one-third of participants. This effect could lead to a substantial impact on education over time, particularly if the disease is left untreated or is recurring, as is frequently the case in endemic settings.^1^ Among adults, activities of daily living were impacted by scabies in approximately one-third of participants, also likely to lead to an impact over time because many communities rely on subsistence agriculture. The presence of scabies did not appear to cause embarrassment, teasing, sexual difficulties or a change the way people dress. This may be due to the high prevalence of the disease in the community, where it is accepted as common, or it may reflect underreporting. Other studies that have reported a greater impact of embarrassment or stigma had a lower overall prevalence of scabies in the community or were conducted among patients presenting to healthcare facilities and thus would be more likely to have troublesome symptoms.^4,6,7^ It was difficult in this field setting to achieve complete privacy for participants when completing the questionnaire, which may have contributed to underreporting. Treatment was rarely reported as having an impact on HRQoL, possibly reflecting the low level of treatment for scabies. Local nurses and participants reported to the study team that the locally available treatment, benzyl benzoate lotion, caused side effects and that treatment was frequently futile because of reinfection from close contacts.

The level of impact of scabies on HRQoL in our study is comparable to previous studies. However, the areas of impact were different, with the greatest impact due to the effects of symptoms. These differences may be explained by the high prevalence and acceptance of scabies in our setting. The community-based nature of the study may have contributed to a lower overall score compared with other studies carried out in clinics where patients actively seek treatment.^6,7^

Neglected tropical diseases that affect the skin may have a wide range of levels of impact. Scabies appears to be at the lower end. For example, studies of filarial lymphoedema on HRQoL showed DLQI mean scores ranging from 2.7 to 10.9.^24,25^ In Ethiopian schoolchildren, both scabies and tungiasis were found to have a similar impact on QoL, with a median CDLQI score of 7 for both diseases.^26^ The minimal clinically important difference (MCID) of the DLQI, or the smallest difference in score that a patient perceives as meaningful and justifies a change in management, has not been established for scabies but is considered to be 4 for inflammatory conditions.^27^ If we accept this MCID of 4, participants in our study with severe scabies would meet this criteria, indicating that a change in management is justified.

We used the standard DLQI and CDLQI questionnaires without modifications. Cross-cultural inequivalence in HRQoL measures should be taken into account when comparing results with other settings.^10^ The sleep domain was a high-scoring domain in the CDLQI in our study, reflecting the effect of scabies-related itch on sleep quality. There is no sleep domain in the DLQI, which may have led to an underestimation of the impact on HRQoL among adults. Modified versions of the DLQI and CDLQI that are relevant to scabies and to cultural setting have been used in other studies.^4,6^ A modified version of the instrument may better reflect the impact of scabies on HRQoL in this setting if it covered the gaps we have identified. Further studies to measure the HRQoL of scabies in Pacific Island countries could consider modifying the instrument in the following ways: to capture the impact of symptoms on sleep among adults, which was a high-scoring domain among children; to better measure healthcare-seeking behaviour in relation to locally available treatment, which may not have been captured because of the way the treatment question was worded; to more appropriately measure the impact on work as it relates to a subsistence lifestyle, which may have been ambiguous in the way work, housework and gardening were differentiated; and to adapt the personal relationships domain in a culturally appropriate way.

There are limitations to our study. First, the control group used to establish a construct validity for this population group was participants with no skin lesions detected on clinical examination. This examination was limited to exposed areas of the body (hands and arms, legs and feet) and a focussed history, and it is possible that some skin conditions were not identified. Second, the questionnaire was administered verbally. A self-administered questionnaire may be answered more honestly but was not possible due to low literacy.^28^ Third, our results reflect the direct impact of scabies and impetigo infestation and do not incorporate the impact of more severe secondary complications and therefore may underestimate the impact. Finally, our study did not assess HRQoL in children <4 y of age, a group with the highest prevalence of scabies.^18^ Although designed for investigating dermatitis, future studies may consider using the Infants Dermatitis IDQOL or FDLQI.^29,30^

Our study demonstrates that scabies has a small, but measurable, impact on HRQoL in Solomon Islands. We have established a construct validity for the use of the DLQI and CDLQI in this region that may extend to other Pacific Island populations. We observed impacts on school, work and gardening that, in this setting, impact an individual's education, future prospects and ability to support their family. In a setting where the population prevalence of scabies is 20%, this accounts for a large impact on the community. There is scope to develop a modified DLQI and CDLQI for scabies that is more culturally appropriate for Pacific Island countries and may better measure the true impact of this disease. The results of this study provide further evidence of the need for elimination of scabies as a public health problem in Solomon Islands and other settings where the disease is endemic.

## Supplementary Material

trab096_Supplemental_File

## Data Availability

The data underlying this article will be shared on reasonable request to the corresponding author.
