# Supplementary material for: Health-related quality of life impact of scabies in the Solomon Islands
Source: Trans R Soc Trop Med Hyg. 2021 Jun 28;116(2):148–56. doi: 10.1093/trstmh/trab096 (PMC8804887; doi:10.1093/trstmh/trab096)
Supplement: trab096_Supplemental_File [file trab096_supplemental_file.docx]

## Supplementary

## Table S1.1. Mean score by life domain for participants with scabies - CDLQI

| Domains | Question | Mean score (95% CI) |
| --- | --- | --- |
| Symptoms and feelings | 1. Over the last week how itchy, “scratchy”, sore or painful has your skin been? | 1.06 (0.90-1.22) |
|  | 2. Over the last week, how embarrassed or self-conscious, upset or sad have you been because of your skin? | 0.04 (0.00-0.08) |
| Personal relationships | 3. Over the last week, how much has your skin affected your friendships? | 0.17 (0.10-0.25) |
| Leisure | 4. Over the last week, how much have you changed or worn different or special clothes/shoes because of your skin? | 0.14 (0.07-0.20) |
|  | 5. Over the last week, how much has your skin affected any going out, playing or doing hobbies? | 0.17 (0.10-0.25) |
|  | 6. Over the last week, how much have you avoided swimming or other sports because of your skin trouble? | 0.14 (0.07-0.20) |
| School or holidays | 7.1. Last week was it school time or holiday time? | School: 84%  Holidays: 16% |
|  | 7.2. If school time: Over the last week how much did your skin problem affect your school work? | 0.56 (0.40-0.72) |
|  | 7.3. If holiday time: How much over the last week, has your skin problem interfered with your enjoyment of the holiday? | 0.06 (0.0-0.20) |
| Personal relationships | 8. Over the last week, how much trouble have you had because of your skin with other people calling you names, teasing, bullying, asking questions or avoiding you? | 0.07 (0.02-0.12) |
| Sleep | 9. Over the last week, how much has your sleep been affected by your skin problem? | 0.50 (0.38-0.65) |
| Treatment | 10. Over the last week, how much of a problem has the treatment for your skin been? | 0.03 (0.0-0.06) |

### Each question scores a maximum of 3 points, question 7.1 is a proportion of respondents and is not included in the score

## Table S1.2. Mean score by life domain for participants with scabies – DLQI

| Domain | Question | Mean score (95% CI) |
| --- | --- | --- |
| Symptoms and feelings | 1. Over the last week how itchy, sore, painful or stinging has your skin been? | 1.03 (0.85-1.21) |
|  | 2. Over the last week, how embarrassed or self-conscious have you been because of your skin? | 0.13 (0.05-0.21) |
| Daily activities | 3. Over the last week, how much has your skin interfered with you going shopping or looking after your home or garden? | 0.43 (0.30-0.55) |
|  | 4. Over the last week, how much has your skin influenced the clothes you wear? | 0.21 (0.12-0.30) |
| Leisure | 5. Over the last week, how much has your skin affected any social or leisure activities? | 0.31 (0.21-0.40) |
|  | 6. Over the last week, how much has your skin made it difficult for you to do any sport? | 0.26 (0.17-0.36) |
| Work and school | 7. Over the last week, has your skin prevented you from working or studying?  If ‘no’ over the last week how much has your skin been a problem at working and studying? | 0.26 (0.09-0.44)  0.20 (0.0-0.50) |
| Personal relationships | 8. Over the last week, how much has your skin created problems with your partner or any close friends or relatives? | 0.26 (0.16-0.37) |
|  | 9. Over the last week, how much has your skin caused any sexual difficulties? | 0.15 (0.07-0.24) |
| Treatment | 10. Over the last week, how much of a problem has the treatment for your skin been, for example by making your home messy, or by taking up time? | 0.02 (0.0-0.0) |

### Each question scores a maximum of 3 points

## Table S2. Not relevant responses on DLQI for participants with scabies

| Question | Not relevant response n (%) |
| --- | --- |
| 3. | 3 (3.3) |
| 4. | 0 |
| 5. | 1 (1.1) |
| 6. | 2 (2.2) |
| 7. | 73 (80.2) |
| 8. | 0 |
| 9. | 11 (12.1) |
| 10. | 0 |

### Question 1 and 2 do not have the option of a ‘not relevant’ response
